# Supplementary material for: Identification of Indicator Genes for Agar Accumulation in Gracilariopsis lemaneiformis (Rhodophyta)
Source: Int J Mol Sci. 2024 Apr 23;25(9):4606. doi: 10.3390/ijms25094606 (PMC11083068; doi:10.3390/ijms25094606)
Supplement: Supplementary file 1 [file ijms-25-04606-s001.zip › ijms-2955046-supplementary.pdf]

Supplementary Materials- Figure

1.

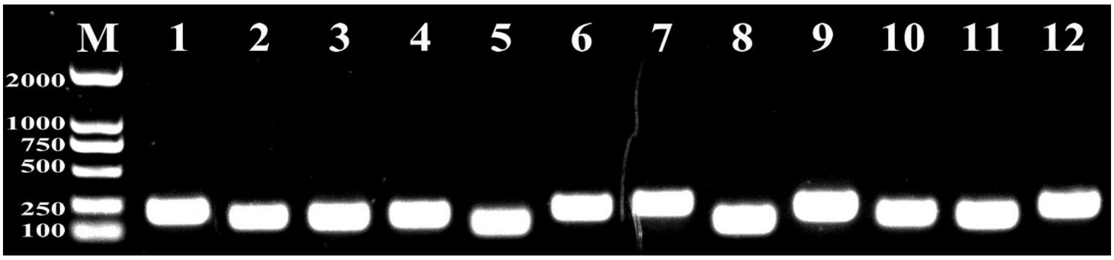

Figure S1 The results of electrophoresis on an agarose gel of the amplification products for the primers (Tm = 55 °C)

Figure S1. lane M: DNA Marker (bp), lane 1: *gpiI*, lane 2: *gpiII*, lane 3: *pgm*, lane 4: *ugp*, lane 5: *galt*, lane 6: *gatI*, lane 7: *gatII*, lane 8: *mpi*, lane 9: *pmm*, lane 10: *mpg*, lane 11: *gme* , lane 12: *act*.

2.

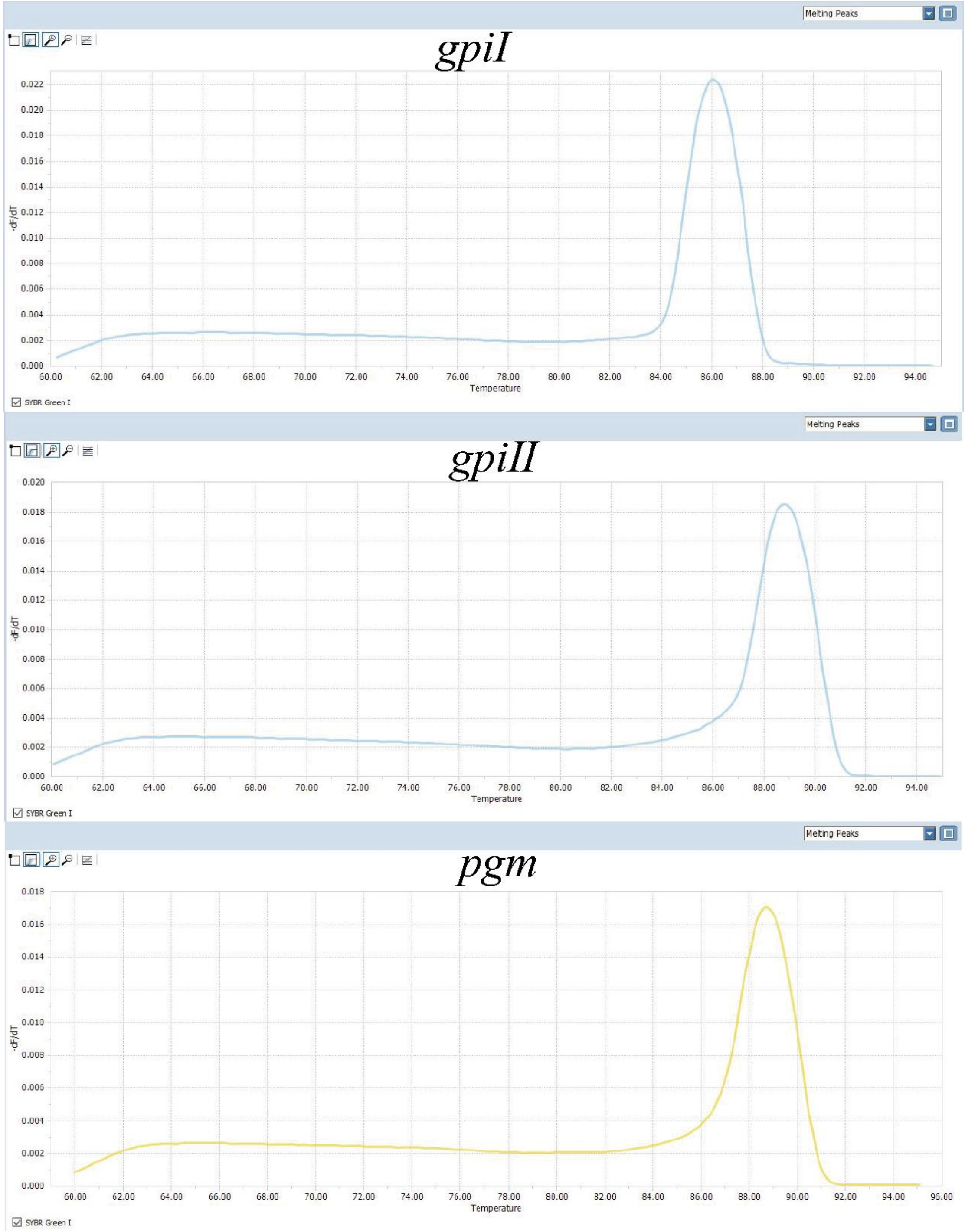

Figure S2 The melting curves of the amplification products for the primers

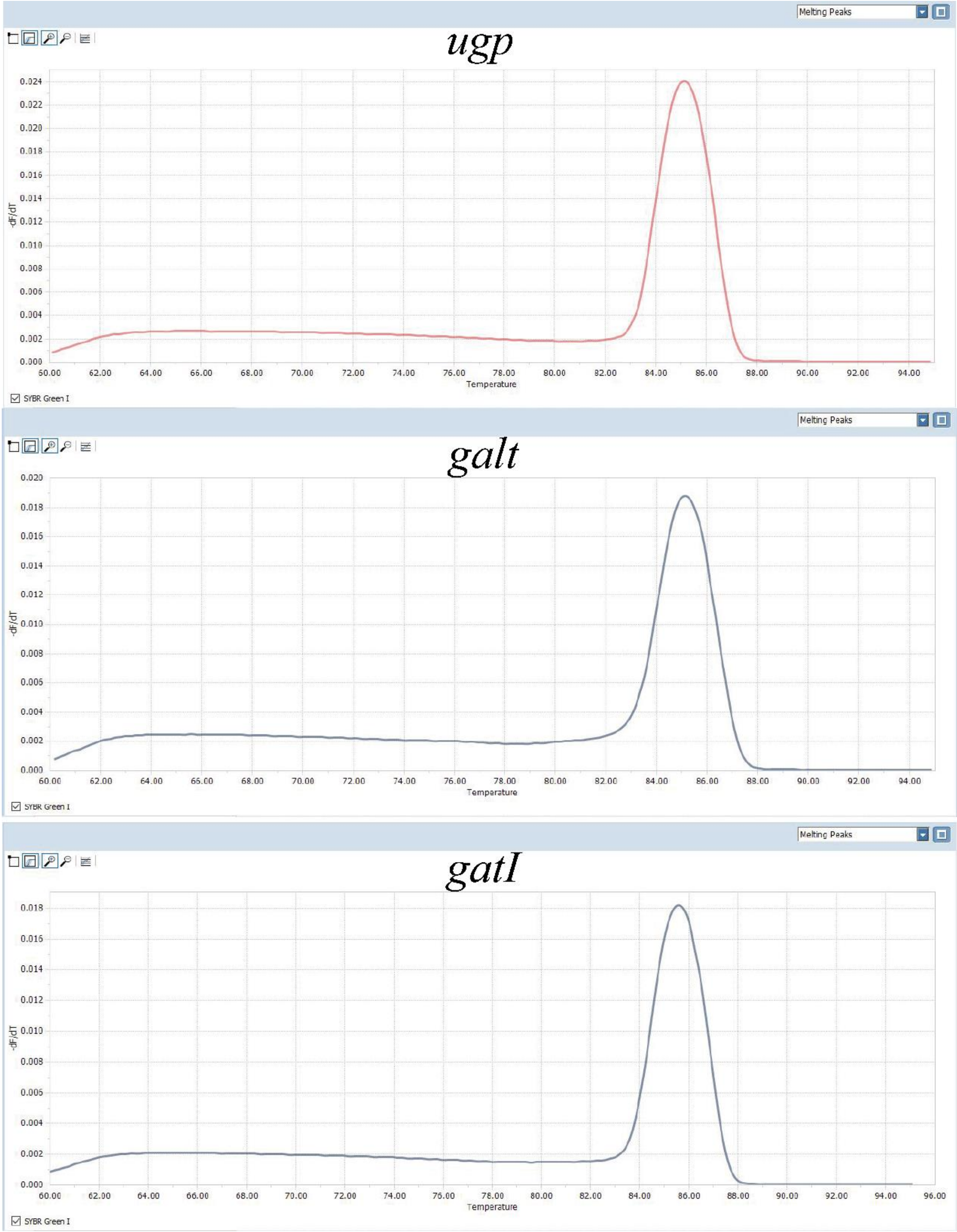

Figure S2 (continued)

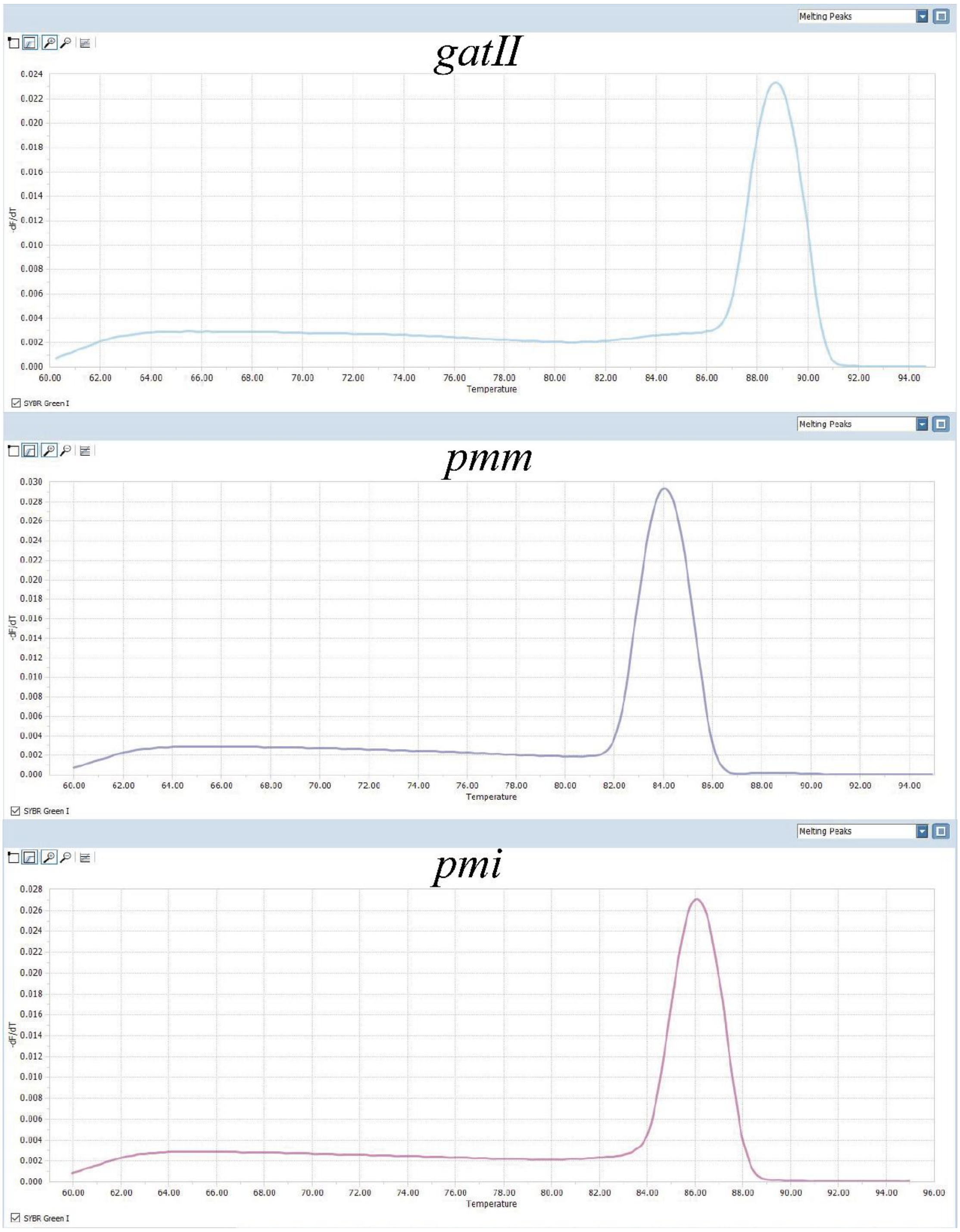

Figure S2 (continued)

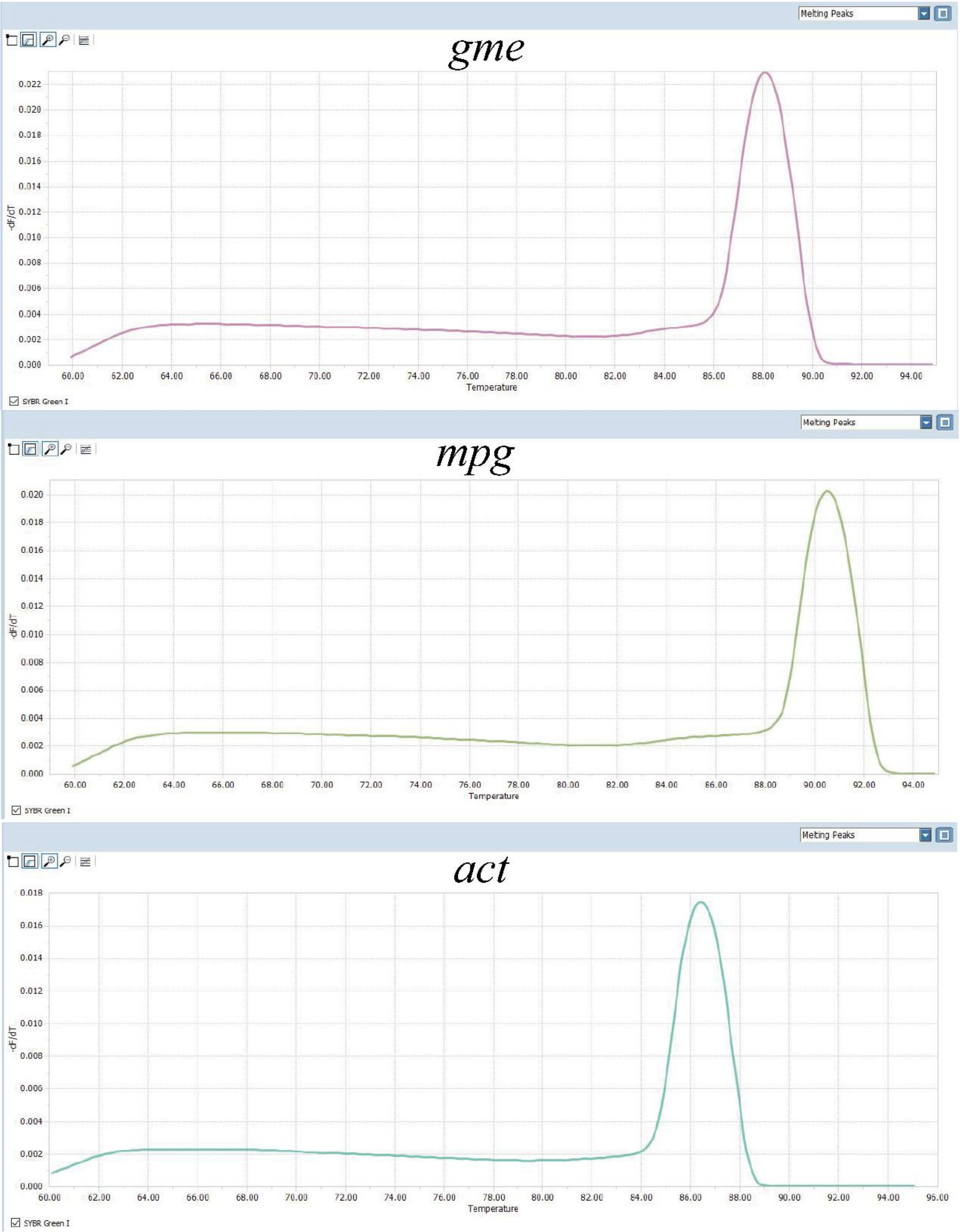

Figure S2 (continued)
